# Using HDR and a template to introduce an in-frame HA tag on the 3′ end of the *Xenopus laevis gata2.L* open reading frame

**DOI:** 10.17912/micropub.biology.000170

**Published:** 2019-09-17

**Authors:** Maya Piccinni, Colin Sharpe, Matt Guille

**Affiliations:** 1 University of Portsmouth, UK

**Figure 1:**
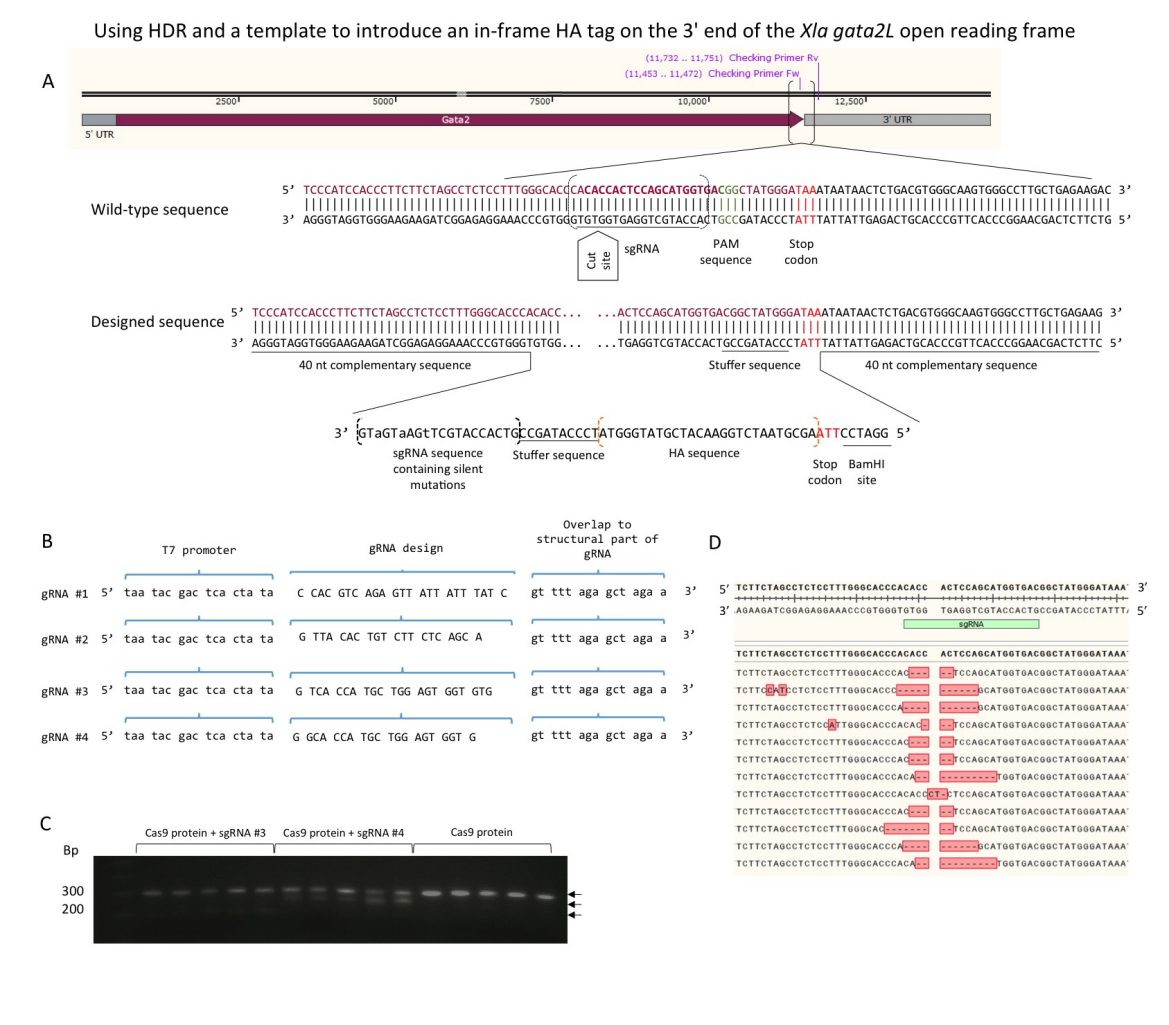
Targeted insertion of an HA tag into the 3’ end of *Xenopus laevis* *gata2.L*. Four single guide RNA (sgRNA) sequences were tested for efficiency by T7 assay before designing the ssOligonucleotide (A) containing the HA tag around the most efficient sgRNA (sgRNA #4, B). The 146 nucleotides long ssDNA fragment contained forty base complementary regions flanking the cutting site containing silent mutations to prevent re-cutting, an HA tag, a stop codon and a BamHI site, to allow the identification of positive clones. Amplification with forward (5’ ACTCCCATCCACCCTTCTTC 3’) and reverse (5’ CGATTGCTGTGTCCGACTTT 3’) primers, followed by digestion of the target region with BamHI would cut the target sequence containing the insertion into two smaller bands that could later be visualised by agarose gel electrophoresis. This approach was not successful. We hypothesise that the mosaicism in F0 animals subjected to PCR reduced the possibility of successfully visualising the digested product. Two primers extending from the HA tag into the genome were designed (HA Fw Primer 5’ CCCATACGATGTTCCAGATTACGC 3’; HA Rv Primer 5’ GCGTAATCTGGAACATCGTATGGG 3’) to selectively amplify the HA insertion successfully discerning between animals containing the insertion and those not. (C.) Five embryo from each experimental condition and five control embryos were analysed and T7 endonuclease treated amplicons were visualized via gel electrophoresis, sub-cloned into pGemTeasy and sequenced (D). Eighty-one percent of the sequences obtained showed deletions around the desired cut site and 6 % contained random insertions.

## Description

Protein detection is a powerful technique used broadly in research, however the successful use of antibodies has its practical limitations presented by the lack of knowledge about the protein in question, poor accessibility of the target sites, specificity barrier and cross-reactivity with other proteins (Fritze and Anderson, 2000). In these cases the insertion of a known short amino acid sequence into the desired locus can be considered. This sequence allows the protein in question to be traceable and detectable by a pre-existing commercial well-characterized antibody. Since the first ever tag was developed in 1943 (Rawson, 1943) there have been many scientific and technological advances which allowed the development of the clustered, regularly interspaced, short palindromic repeats (CRISPR) technology which we use here to insert an HA tag (YPYDVPDYA) into the 3’ end of the *Xenopus laevisgata2.L*gene (NM_001090574.1).

Four single guide RNAs were designed to anneal to a particular sequence in the genome (*Xenopus laevis*J strain 9.1), guiding Cas9 to make the double-stranded cut 3 bp away from the protospacer adjacent motif (PAM) site (Mojica *et al*., 2009)close to the stop codon of the *Xenopus laevis gata2.L*gene (Fig1A). The sgRNA sequence that anneals to the target sequence is 20 nucleotides long with the T7 polymerase promoter sequence at the 5’ and a 15 nucleotide sequence at the 3’ end that anneals to a standard oligonucleotide (Nakayama *et al.*, 2013)(Fig1B), which in turn forms a secondary structure that binds the Cas9 protein. In this study, CRISPRscan (Moreno-Mateos *et al.*, 2015),an online predictive sgRNA-scoring algorithm, was used to identify potential sgRNAs and the highest scoring sgRNA were used.

The sgRNAs were synthesised as previously described (Nakayama *et al.*, 2013). In summary, the synthetic sgRNA encoding sequences were annealed to a universal single-stranded oligonucleotide and filled in using Taq polymerase to make a double-stranded DNA. The resulting amplicons were visualized by gel electrophoresis and RNA synthesized using Megashortscript™ T7 Transcription Kit (Invitrogen) following the manufacturer’s instructions; the RNA product was then visualised on a 1.5 % agarose gel.

To test which was the most efficient sgRNA, three amounts of each sgRNA were tested, 50 pg, 100 pg and 250 pg; a 4 µl mix was prepared containing 4 µM Cas9 protein, the chosen sgRNA amount and dH_2_O. Embryos were injected at the 1-cell stage with 4 nl of the mix and allowed to develop to stage 16. The embryos injected with 250 pg of the sgRNA failed to gastrulate and survivors were deformed. All the following injections were thus made using 100 pg of sgRNA. Genomic DNA was extracted from 20 individual embryos and the target region amplified by PCR. To test if the cas9/sgRNA injections successfully altered the target gene the amplicons were denatured and allowed to reanneal. Once annealed, T7 endonuclease was introduced into the mix and further incubated. The reaction products were then visualised via gel electrophoresis; homoduplexes are not cut by the enzyme and heteroduplexes are, a difference readily detected by gel electrophoresis. The stronger the lower (digested) band the more efficient the sgRNA is. Three of the sgRNAs produced very weak lower bands, one cut more than half of the amplicon. This sgRNA was used for all further experiments.

To assess the efficiency and specificity of the mutagenesis, the target PCR products were sub-cloned into pGemTeasy and sequenced. Eighty-one percent of the sequences obtained showed deletions around the desired cut site. It also showed some random insertions in 6 % of the sequenced clones (Fig1C; Fig1D).

Embryos were co-injected at the one-cell stage with sgRNA, Cas9 Nuclease NLS (S. pyogenes, NEB) and ssOligonucleotide (Fig1A), and allowed to grow. Cas9 protein was injected on its own as a negative control. The majority of embryos did not develop after gastrulation and were mostly deformed with most of the deaths between stages 8 and 12, correlating with the onset of expression of *gata2.L*in the embryo. Tadpoles were tested by PCR of the extracted genomic DNA and positive PCR amplicons were cloned into pGemTeasy for sequencing. From each cloning reaction, 5 colonies were prepped and sequenced. The sequences showed a variety of in-frame and out-of-frame insertions. From over 6000 injected embryos, 168 (2.8 %) grew to tail-bud stage from which 27 of the F0s tested positive for the in-frame insertion (0.45 %).

Fifteen frogs developed to adulthood, 8 (0.13 % of the original 6000) of these produce offspring containing the in-frame HA tag. Screening of F1 embryos is ongoing, with the aim of detecting the Gata2.L-HA protein by western blot and immunohistochemistry.

The extremely low insertion rate can be attributed to non-homologous end joining (NHEJ) background and probably the competition between homology directed recombination (HDR) and replication which is very rapid in the early embryo, generating highly mosaic embryos. One route that can overcome these issues, and thus increase the insertion rate and lower the embryo mosaicism is to modify the oocyte before fertilization. Aslan et al (2017) have shown that by injecting the CRISPR components into oocytes followed by host transfer that they increase the rates of HDR over NHEJ, producing non-mosaic embryos.

## Reagents

Cas9 Nuclease NLS, S. pyogenes (NEB)

pGEM®-T Easy Vector Systems (Promega)

E. coli DH5α (homemade)

GoTaq® G2 Hot Start Master Mixes (Promega)

Megashortscript™ T7 Transcription Kit (Thermo Fisher)
